# Supplementary material for: Assessment of Body Condition in a Threatened Dolphin Population in an Anthropized Area in Southeastern Brazil
Source: Animals (Basel). 2024 Jun 26;14(13):1887. doi: 10.3390/ani14131887 (PMC11240347; doi:10.3390/ani14131887)
Supplement: Supplementary file 1 [file animals-14-01887-s001.zip › animals-2966915-supplementary.pdf]

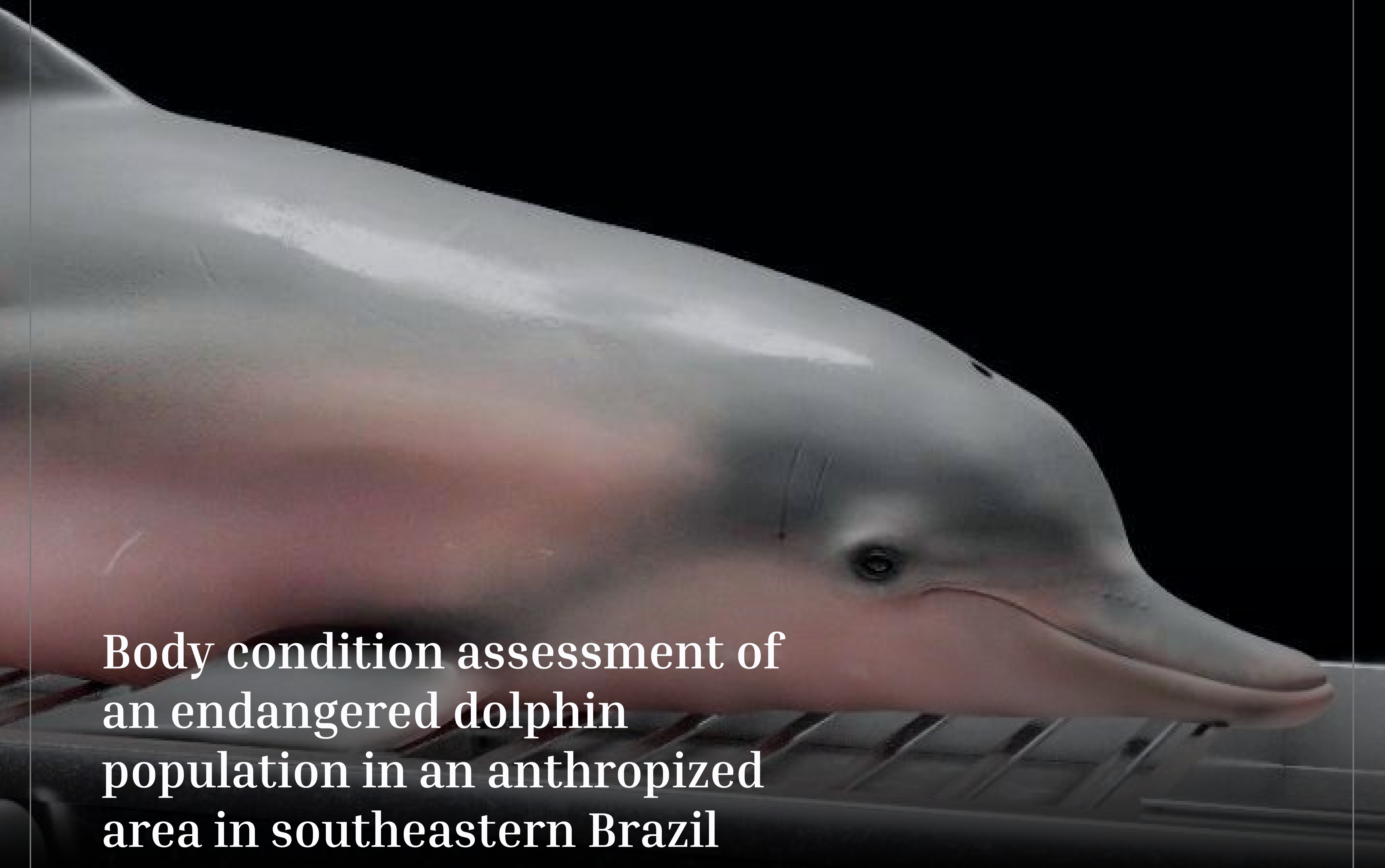

Body condition assessment of an endangered dolphin population in an anthropized area in southeastern Brazil

Deyverson Silva*[1], Guilherme Maricato[1,2,3], Tomaz Cezimbra[1], Larissa Melo[1], Israel S. Maciel[1,2], Rodrigo Tardin[1]

Body Condition Score: 1 [Good]

Not all parameters can be present in all animals due to the particularities of each individual

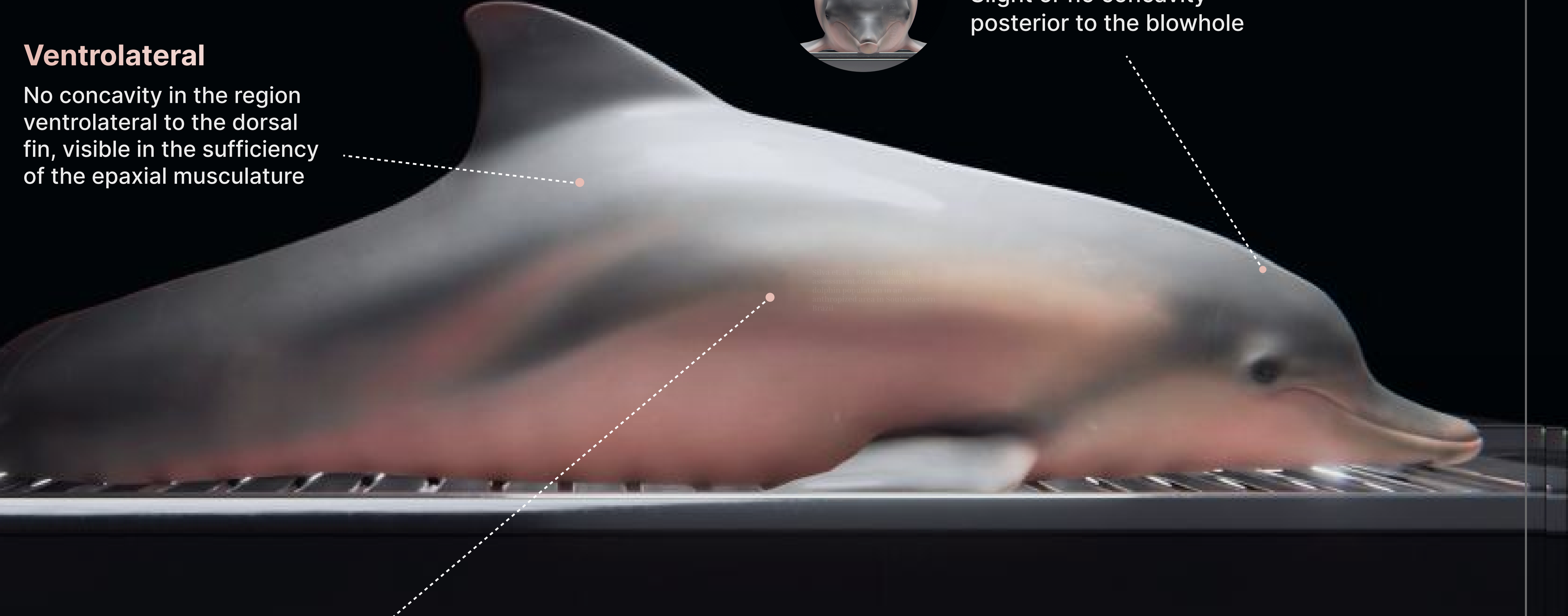

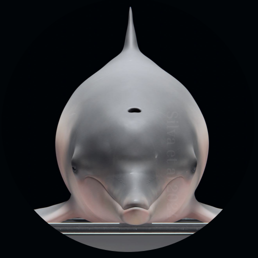

Blowhole

Slight or no concavity posterior to the blowhole

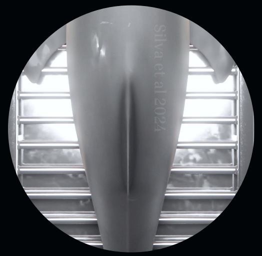

Ventrolateral

No concavity in the region ventrolateral to the dorsal fin, visible in the sufficiency of the epaxial musculature

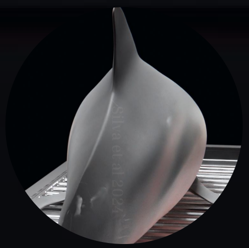

Body

Normal-looking and/or rounded body with no visible evidence of thinness

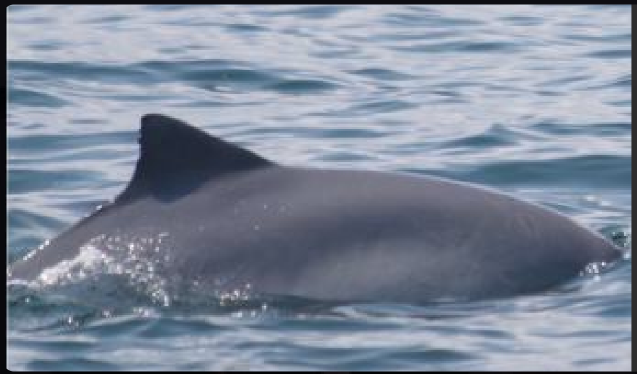

Fundamental informations

Monitoring changes in the body condition of cetaceans can provide fundamental information on how populations express themselves in the face of prey availability, pre and post-reproductive events, and how these are linked to population conservation status [8]

Body Condition Score: 2 [Thin]

Not all parameters can be present in all animals due to the particularities of each individual

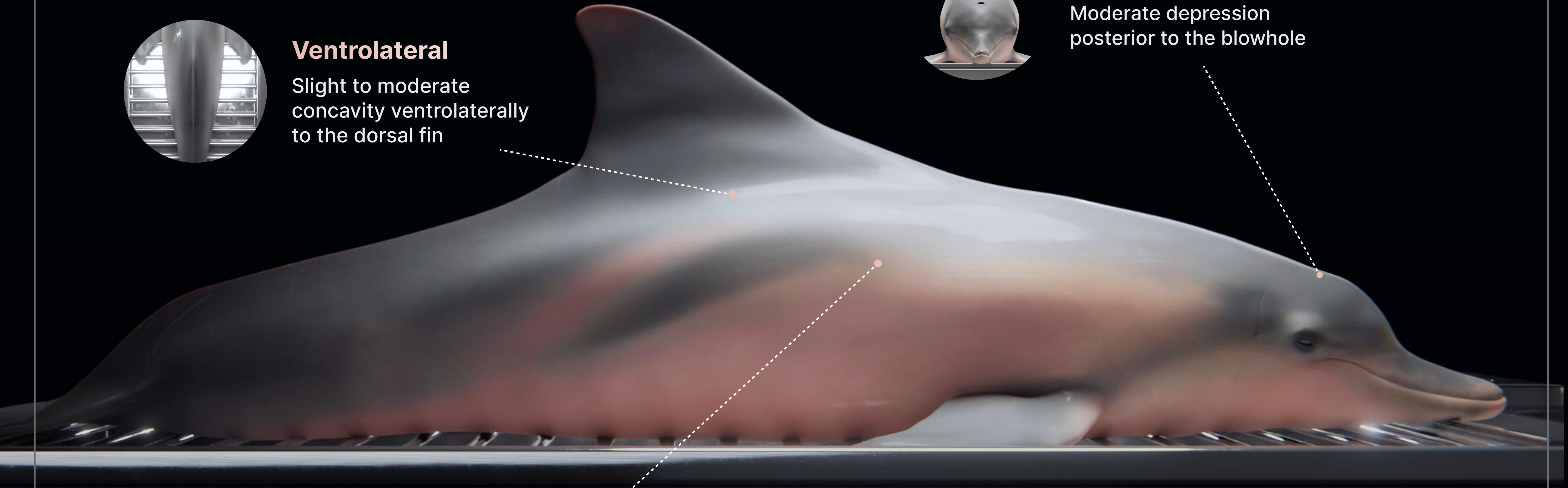

Ventrolateral

Slight to moderate concavity ventrolaterally to the dorsal fin

Blowhole

Moderate depression posterior to the blowhole

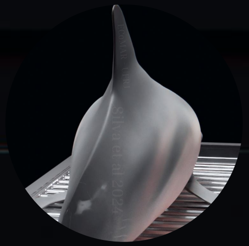

Body

Slightly thin body, with absent or barely visible bone structures

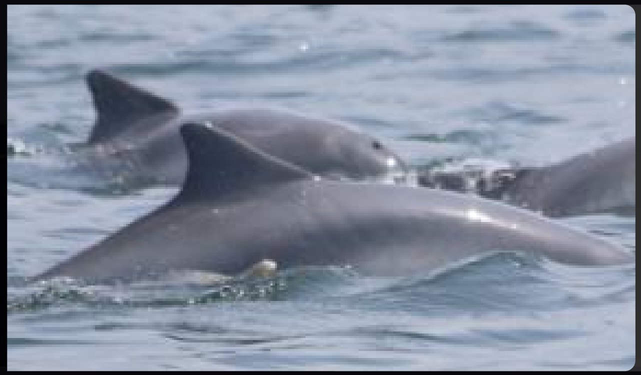

Fundamental informations

The study's findings highlight a population with more than half of the sampled individuals in poor body condition that inhabits a region suffering from multiple human cumulative impacts.

Body Condition Score : 3 [Emaciated]

Not all parameters can be present in all animals due to the particularities of each individual

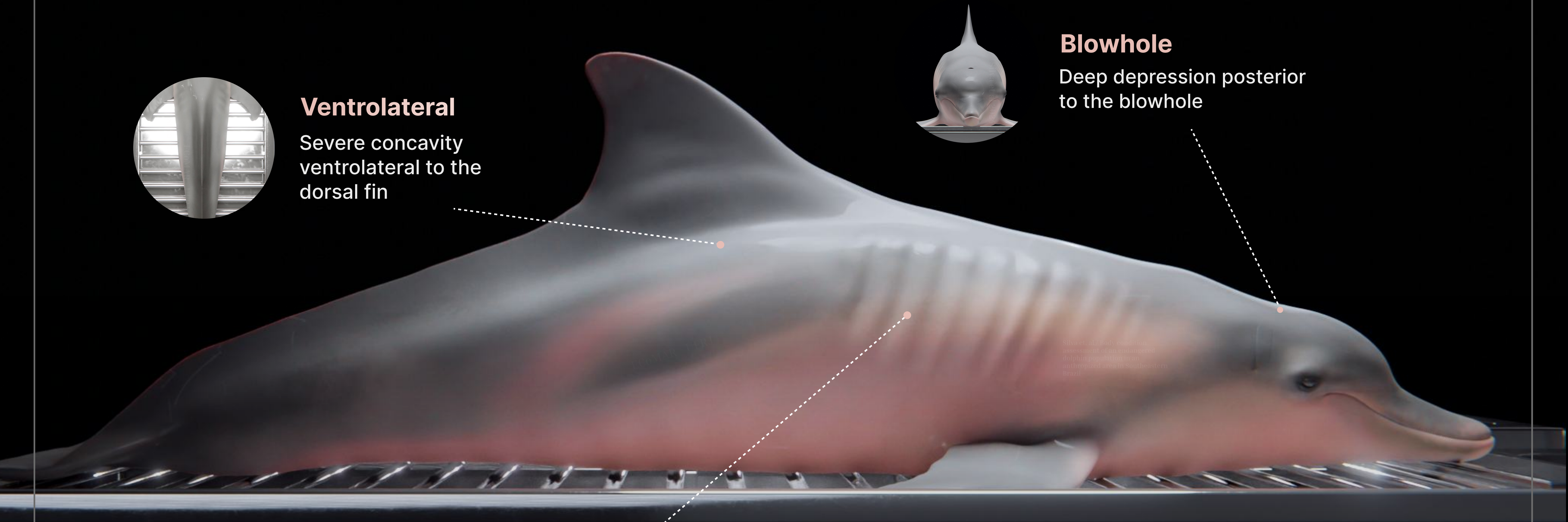

Ventrolateral

Severe concavity ventrolateral to the dorsal fin

Blowhole

Deep depression posterior to the blowhole

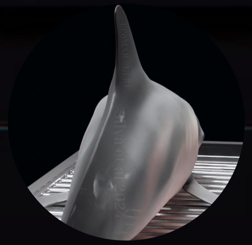

Body

Evident loss of muscle mass and severe or moderate visibility of the rib bones

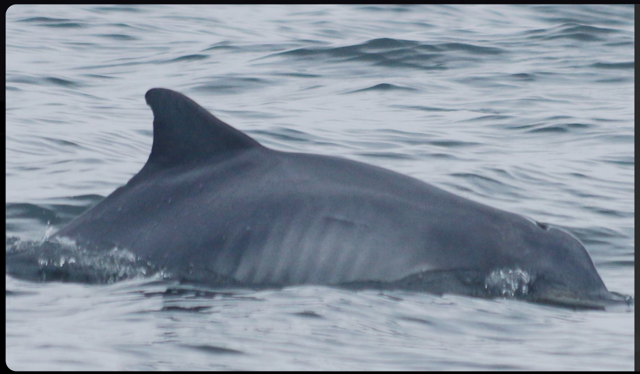

Fundamental informations

The presence of commercial ships, fishing, and tourist boats in areas where food is available can negatively affect the body condition of Guiana dolphins and put them at increased health risk.

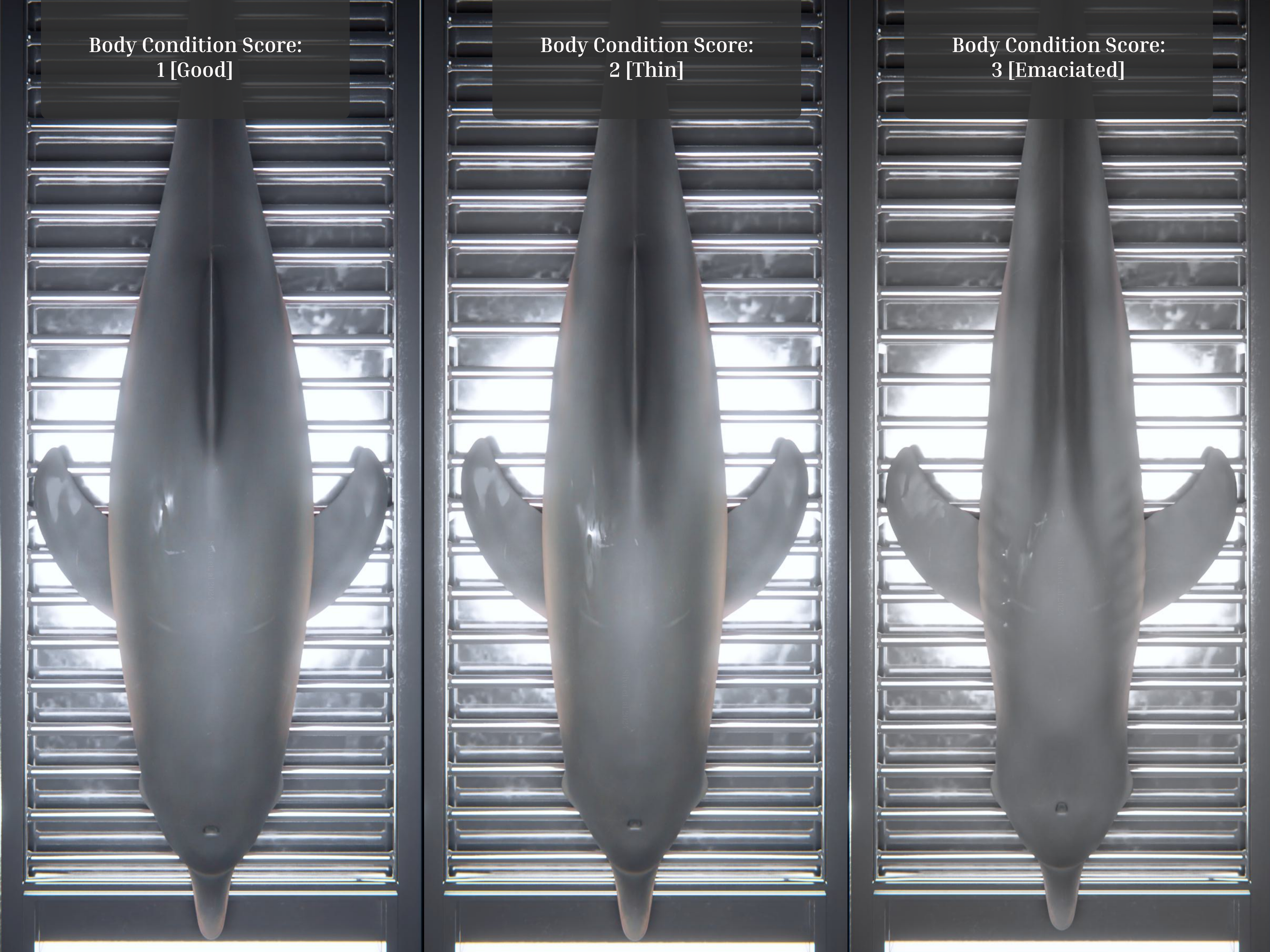
Body Condition Score:
1 [Good]
Body Condition Score:
2 [Thin]
Body Condition Score:
3 [Emaciated]

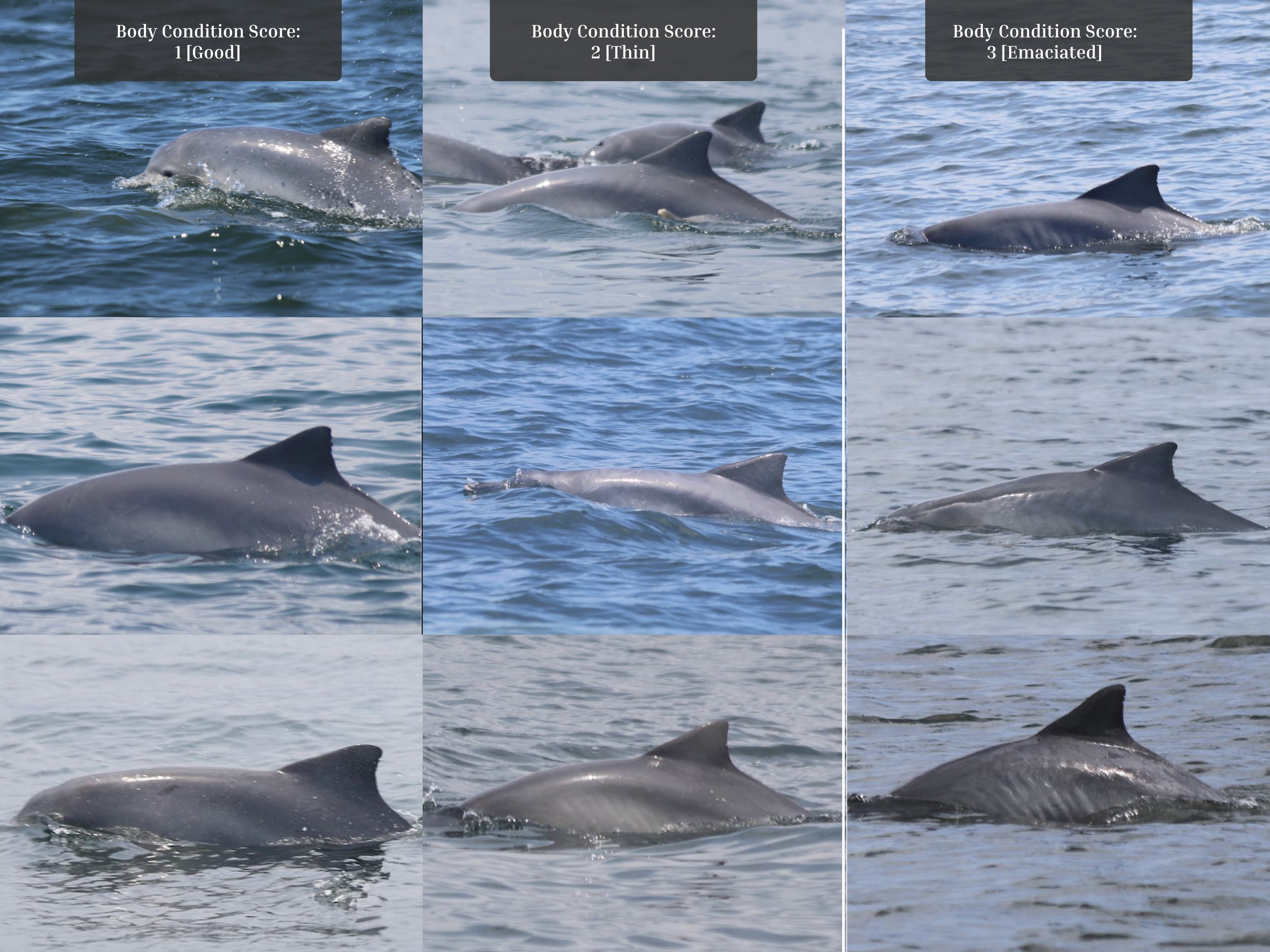
Body Condition Score:
1 [Good]
Body Condition Score:
2 [Thin]
Body Condition Score:
3 [Emaciated]

Body condition assessment of an endangered dolphin population in an anthropized area in southeastern Brazil

Deyverson Silva*[1], Guilherme Maricato[1,2,3], Tomaz Cezimbra[1], Larissa Melo[1], Israel S. Maciel[1,2], Rodrigo Tardin[1]

Corresponding author: deyversonbio@outlook.com (Deyverson Silva)
